# Admission hyperglycemia and outcome after intravenous thrombolysis: is there a difference among the stroke-subtypes?

**DOI:** 10.1186/s12883-016-0617-0

**Published:** 2016-07-15

**Authors:** Irene Miedema, Gert-Jan Luijckx, Raf Brouns, Jacques De Keyser, Maarten Uyttenboogaart

**Affiliations:** Department of Neurology, University Medical Center Groningen, University of Groningen, Hanzeplein 1, 9700 RB Groningen, The Netherlands; Department of Neurology, Universitair Ziekenhuis Brussel, Center for Neurosciences (C4N), Vrije Universiteit Brussel (VUB), Brussels, Belgium

**Keywords:** Acute ischemic stroke, Hyperglycemia, Functional outcome, Tissue plasminogen activator, Stroke subtype

## Abstract

**Background:**

The prognostic influence of hyperglycemia in acute stroke has been well established. While in cortical stroke there is a strong association between hyperglycemia and poor outcome, this relation is less clear in lacunar stroke. It has been suggested that this discrepancy is present among patients treated with intravenous tissue plasminogen activator (tPA), but confirmation is needed.

**Methods:**

In two prospectively collected cohorts of patient treated with intravenous tPA for acute ischemic stroke, we investigated the effect of hyperglycemia (serum glucose level >8 mmol/L) on functional outcome in lacunar and non-lacunar stroke. Poor functional outcome was defined as modified Rankin Scale score ≥ 3 at 3 months.

**Results:**

A total of 1012 patients was included of which 162 patients (16 %) had lacunar stroke. The prevalence of hyperglycemia did not differ between stroke subtypes (22 % vs 21 %, *p* = 0.85). In multivariate analysis hyperglycemia was associated with poor functional outcome in non-lacunar stroke (OR 2.1, 95 % CI 1.39–3.28, *p* = 0.001). In patients with lacunar stroke, we did not find an association (OR 1.8, 95 % CI 0.62–4.08, *p* = 0.43).

**Conclusion:**

This study confirms a difference in prognostic influence of hyperglycemia between non-lacunar and lacunar ischemic stroke.

## Background

Admission hyperglycemia is common in patients with acute ischemic stroke and occurs among all ischemic stroke subtypes [1]. Acute ischemic stroke can give rise to abnormalities in glucose metabolism inducing a stress hyperglycemia, also in non-diabetic patients [2]. Admission hyperglycemia is strongly associated with poor functional outcome after ischemic stroke, regardless of a history of diabetes [3–10]. In patients treated with intravenous recombinant tissue plasminogen antigen (tPA), hyperglycemia was associated with lower recanalization rates, poor functional outcome, higher mortality and increased risk of symptomatic intracerebral hemorrhage (SICH) [3–10].

**Fig. 1:**
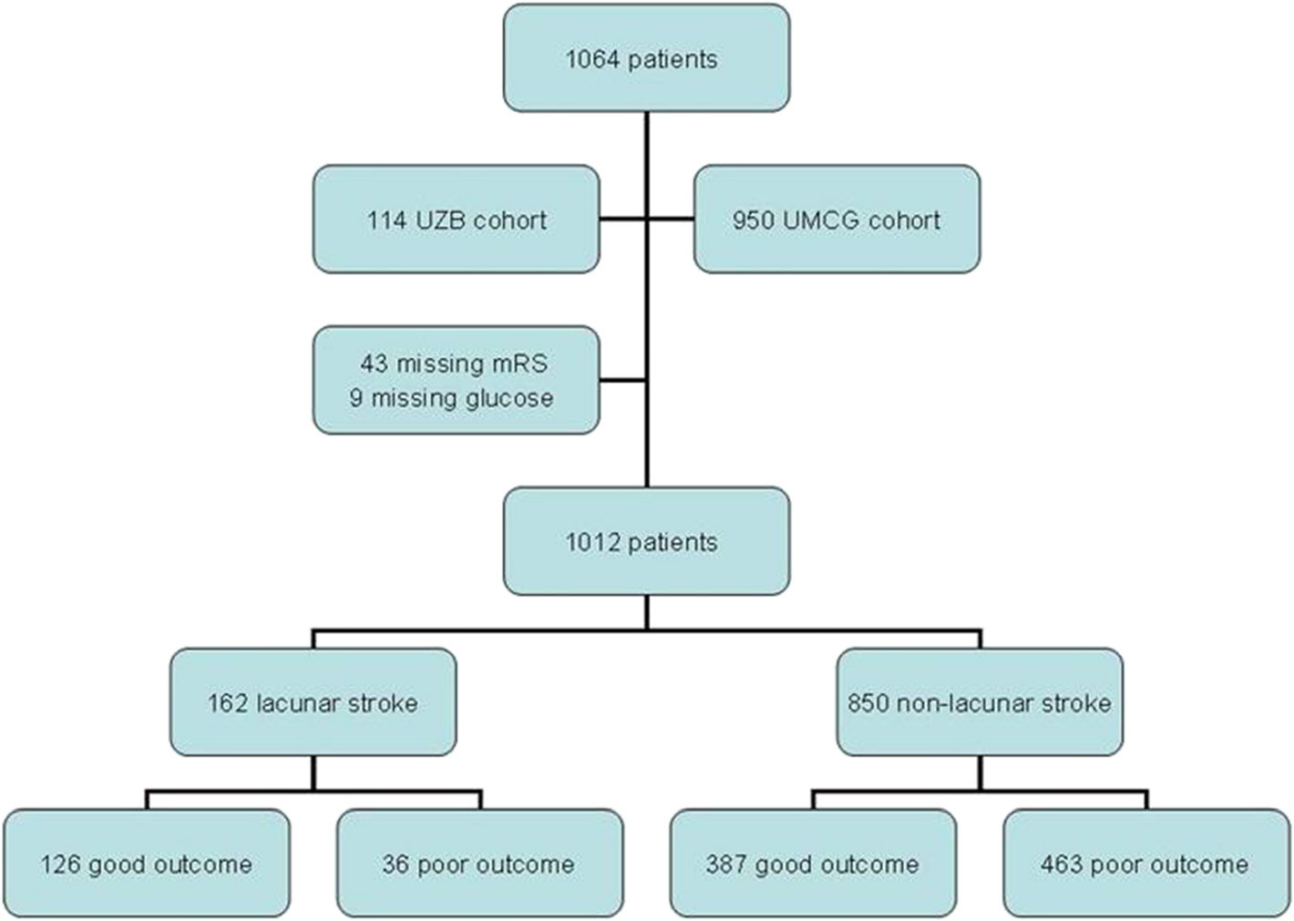
Flowchart with patient characteristics

Two previous studies found that the prognostic value of admission hyperglycemia differed among ischemic stroke subtypes. Hyperglycemia was associated with poor outcome in patients with large vessel stroke, but a favorable effect of moderate hyperglycemia was suggested in lacunar stroke [7, 9]. Both studies investigated patients that did not receive thrombolytic therapy. Hyperglycemia is thought to augment reperfusion injury by increased oxidative stress and inflammatory respons [11]. Furthermore, in large vessel stroke hyperglycemia was associated with reduced salvage of the penumbra and larger infarct size [6].

It could be expected that in patients treated with intravenous tPA the difference in functional outcome between these two stroke subtypes would be even more striking, because hyperglycemia is associated with reperfusion injury. Moreover this information could be used to guide the setup of additional clinical trials investigating the role of blood glucose lowering therapy in tPA treated patients. Recently, a post-hoc analysis of the NINDS dataset confirmed this hypothesis [12]. Mandava *et al.* showed that hyperglycemia was associated with worse functional outcome in patients with large vessel occlusive stroke, where as in small vessel occlusive stroke no difference in outcome between normoglycemic and hyperglycemic patients was found. However, extrapolation of these results may be hampered by the small patient group sizes. We aimed to investigate the effect of admission hyperglycemia on functional outcome in a large cohort of patients with lacunar and non-lacunar stroke treated with intravenous tPA. We tested the hypothesis that admission hyperglycemia is a negative predictor of functional outcome in non-lacunar stroke but not in lacunar stroke.

## Methods

Data were obtained from two stroke centers in the Netherlands and Belgium. Both centers have an ongoing prospective registry of consecutive patients with acute ischemic stroke receiving intravenous tPA treatment. The registry was started at the University Medical Center of Groningen (UMCG) in April 2002. All patients registered between April 2002 and December 2013 were included in this study. In the Universitair Ziekenhuis Brussel (UZB), the registry was started in March 2009. In both hospitals, tPA treatment was performed within a time window of 4.5 h after onset of symptoms according to a protocol which has been described earlier [13]. According to the local ethical boards criteria, no ethical approval was required to use the anonymized data in the registries.

Stroke severity before administration of tPA was assessed according to the National Institute of Health Stroke Scale (NIHSS). The stroke subtype was classified as lacunar stroke (LACI = lacunar infarct) or non-lacunar stroke (PACI = partial anterior circulation infarct, TACI = total anterior circulation infarct and POCI = posterior circulation infarct) according to the Oxfordshire Community Stroke Project Classification (OCSP) [14].

Demographic and clinical information was recorded, including cardiovascular risk factors and serum glucose concentration. Blood glucose levels were determined in a venous blood sample on admission at the emergency room, before tPA treatment. We decided to assess the relation between admission hyperglycemia and outcome, because the (non-fasting) blood glucose levels during the acute phase are most likely to influency the ischemic penumbra in acute stroke. Hyperglycemia was defined as >8 mmol/L (≈144 mg/dl) in accordance with previous studies on this subject [5, 8, 12, 15].

### Outcome

The modified Rankin Scale (mRS) was used to determine functional outcome at 3 months after stroke onset. Functional outcome was dichotomized into poor outcome meaning dependence or death (mRS 3–6) and favorable outcome (mRS 0–2), corresponding to independence with regard to activities of daily living [16]. This cut-off score was preferred as it allows comparison with previous reports on this topic [5, 7, 8]. Outcome was assessed by experienced stroke nurses.

### Statistics

Baseline characteristics for patients stratified by stroke subtype were compared. Mann Whitney U-test was used for continuous and ordinal variables without a normal distribution. Pearson’s Chi Square test and Fisher’s exact test were used for dichotomous variables. All factors with a p-value <0.20 in baseline characteristics were also added to the initial multivariate analysis. In the multivariate analysis, age and baseline NIHSS score were added as covariates because these variables are generally accepted as independent predictors of stroke outcome [17]. Given the aim of the study, a history of diabetes was also entered in the multivariable analysis and an interaction-term between hyperglycemia and a history of diabetes was tested. In the multivariate analysis variable with a p value > 0.20 were removed from the final model. All statistical analyses were performed using PASW Statistics 20.0. Statistical significance is taken to be at two tailed level < 0.05. A binary logistic regression model is used for multivariable analysis, with adjustment for possible confounders, to calculate odds ratios with 95 % confidence interval.

## Results

One thousand and sixty-four patients were treated with tPA during the study period. Three month mRS scores of 43 patients were missing (4 % of total population), 30 patients of the UMCG cohort (3 %) and 13 patients of the UZB cohort (11 %). These patients were excluded from the analysis. Of the remaining 1021 patients nine patients (1 %) were excluded because of missing data on stroke subtype according to the Oxfordshire Community Stroke Project Classification. We included a total of 1012 patients consisting of 162 patients (16 %) with lacunar stroke and 850 patients (84 %) with non-lacunar stroke. In the non-lacunar stroke group, 480 patients (47 %) had PACI, 297 patients (29 %) TACI and 73 patients (7 %) POCI.

The baseline characteristics are presented in Table 1. Patients with lacunar stroke were significantly younger than patients with non-lacunar stroke and they had lower baseline NIHSS. Patients with lacunar stroke were more often current smokers and more frequently had a history of hypercholesterolemia. As expected, presence of atrial fibrillation was less common in patients with lacunar stroke. No difference in baseline serum glucose levels were found between the two stroke subtypes (6.9 mmol/L versus 6.9 mmol/L, *p* = 0.78) and the occurrence of admission hyperglycemia, did not differ between the subtypes (22 % versus 21 %, *p* = 0.85). The onset to treatment was higher in lacunar stroke.

**Table 1:** Baseline characteristics

	Lacunar stroke	Non-lacunar stroke	
Characteristic	*N* = 162	*N* = 850	*p*-value
Male (%)	94 (58 %)	459 (54 %)	0.35
Mean age (SD), years	66 (13)	69 (14)	0.004^d^
Median NIHSS at presentation (IQR)	7 (5–9)	12 (7–17)	<0.001^d^
Mean serum glucose level (SD), mmol/L	6.9 (2.1)	6.9 (2.3)	0.78^d^
Mean onset to treatment time (SD), minutes^a^	164 (81)	151 (58)	0.04^c^
Hyperglycemia (>8 mmol/L)	35 (22 %)	178 (21 %)	0.85
Total cholesterol (SD) mmol/L^b^ HDL (SD) mmol/L^b^ LDL (SD) mmol/L^b^	4.7 (1.9) 1.4 (0.6) 2.9 (1.4)	4.5 (1.9) 1.1 (0.6) 2.8 (1.4)	0.36 ^d^ 0.61 ^d^ 0.55 ^d^
Vascular risk factors			
Hypertension (%)	84 (52 %)	426 (50 %)	0.69
Diabetes (%)	32 (20 %)	128 (15 %)	0.13
Hypercholesterolemia (%)	103 (64 %)	460 (54 %)	0.026
Atrial fibrillation (%)	22 (14 %)	201 (24 %)	0.005
Smoking (%)^c^	53 (33 %)	206 (25 %)	0.04

Values are number unless otherwise indicated

*IQR* inter-quartile range, *SD* standard deviation, *HDL* high density lipoprotein, *LDL* low density lipoprotein. ^a^115 missing, ^b^ 248 missing, ^c^ 41 missing. *P* values calculated with Pearson’s χ^2^-test, unless otherwise indicated. ^d^ Mann–Whitney U-test

### Functional outcome

In total, 499 patients (49 %) had a poor outcome (mRS 3–6), see also Fig. 1. The occurrence of poor outcome was significantly different between patients with lacunar and non-lacunar stroke (22 % versus 55 %, *p* = < 0.001).

Univariate analysis showed that patients with lacunar stroke and normoglycemia less frequently had poor outcome than those with hyperglycemia (19 % versus 34 % respectively, *p* = 0.047). After adjustment for possible confounders in multivariate analysis, admission hyperglycemia was not associated with poor functional outcome (OR, 1.8; 95 % CI, 0.62–4.08; *p* = 0.43) (Table 2).

**Table 2:** Multivariate analysis: association of hyperglycemia > 8 mmol/L and functional outcome

	Poor outcome (mRS 3–6)					
	Lacunar stroke			Non-lacunar stroke		
Variables	OR	95 % CI	*p*	OR	95 % CI	*p*
Hyperglycemia, >8 mmol/L	1.58	0.62–4.08	0.34	2.10	1.39–3.28	0.001
Age, year	1.04	1.01–1.07	0.02	1.05	1.03–1.06	<0.001
NIHSS score	1.19	1.06–1.35	<0.001	1.23	1.19–1.27	<0.001
History of diabetes	1.90	0.73–5.03	0.19	1.34	0.83–2.16	0.24

*CI* confidence interval, *mRS* modified Rankin Scale, *NIHSS* National Institutes of Health Stroke Scale, *OR* odds ratio. Hosmer-Lemeshow: 0.71/0.67

In non-lacunar stroke, univariate analysis also showed a significant difference between normoglycemic and hyperglycemic patients with regard to poor outcome (51 % versus 67 %, *p* = <0.001). The association between admission hyperglycemia and poor outcome was confirmed in multivariate analysis taking possible confounders into account (OR, 2.1; 95 % CI, 1.39–3.28; *p* = 0.001) (Table 2). Older age and higher baseline NIHSS score were associated with poor functional outcome in both stroke subtypes (Table 2). Testing for the interaction term including hyperglycemia and diabetes did not change the results in the multivariate models.

### Symptomatic intracerebral hemorrhage

There were 35 (3.5 %) patients in the entire cohort with a symptomatic intracerebral hemorrhage (SICH) after rtPA treatment. There was no significant difference in SICH rate between lacunar and non lacunar strokes (respectively 3/162 (1.9 %) versus 32/850 (3.8 %), *p* = 0.35). Mean admission glucose levels were non signicantly higher in patients SICH compared to patients without SICH (7.4 mmol/L versus 6.8 mmol/L, *p* = 0.78).

## Discussion

Our results show that hyperglycemia independently predicted poor functional outcome, in tPA-treated patients with non-lacunar stroke but not in patients with lacunar stroke. These findings are consistent with the results of the small retrospective post-hoc study of the NINDS dataset [12]. Higher NIHSS scores and older age were associated with poor functional outcome in our study. Both variables have previously been reported as prognostic variables in tPA treated patients [18].

The mechanisms by which hyperglycemia augments ischemic brain injury are not fully understood and several mechanisms are thought to play a role in the detrimental effect. Hyperglycemia may have a detrimental effect in large vessel stroke due to anaerobic glycolysis enhancing acidosis and free radical production, increase of blood–brain barrier permeability, increase of coagulation processes and decreased fibrinolytic activity, and induction of vascular changes with a pro-vasoconstrictive, pro-thrombotic and pro-inflammatory effect which compromises the collateral circulation and enhances reperfusion injury, in the ischemic penumbra [11, 15, 19–25]. The rather favorable functional outcome in patients with lacunar stroke and moderate hyperglycemia may be related to the absence of an ischemic penumbra [6, 7], and anaerobic glycolysis leading to increased astrocytic production of lactate, which is an important source of energy for axons and oligodendrocytes [26, 27].

Furthermore, a correlation was found between higher lactate levels in the cerebrospinal fluid and poor functional outcome in patients with non-lacunar stroke, but not in patients with lacunar stroke [28]. Plausibly, this mechanism may counterbalance the negative effects of hyperglycemia in lacunar stroke. It should be noted, however, that the exact mechanisms why hyperglycemia has a different prognostic influence in lacunar and non lacunar stroke are not elucidated yet, and the these hypotheses have yet to be proven.

Trials investigating the effects of glucose lowering in acute ischemic stroke showed mostly disappointing results. Overall, no improvement of functional outcome was found [29, 30]. Large trials on this subject are ongoing, including patients treated with intravenous tPA. (www.clinicaltrials.gov, e.g. ID NCT01369069).

Our study has several limitations. Despite the quite large sample size, the subgroup of patients with lacunar stroke is relatively small, which may negatively influence the statistical power of our findings. Information on serial glucose measurements or glycosylated hemoglobin concentrations were not available and we did not perform a MRI of the brain to confirm the clinical classification of stroke subtype, as lacunar stroke based on the OCSP classification is not exactly the same as small vessel disease (TOAST criteria). At last, as we did not record variables like antidiabetic medication use or body mass index, we cannot not rule out that these variables could have confounded our results.

## Conclusion

The results of our prospective study confirm the results of an earlier post-hoc analysis on this subject and lend further support for a different approach of hyperglycemia in non-lacunar and lacunar ischemic stroke.

## Abbreviations

CI, confidence interval; IQR, interquartile range; LACI, lacunar infarction; mRS, modified Rankin Scale; NIHSS, National Institute of Health Stroke Scale; NINDS, National Institute of Neurological Disorders and Stroke; OR, odds ratio; PACI, partial anterior circulation infarct; POCI, posterior circulation infarct; SD, standard deviation; SICH, symptomatic intracerebral hemorrhage; TACI, total anterior circulation infarct; tPA, tissue plasminogen activator; UMCG, University Medical Center Groningen; UZB, Universitair Ziekenhuis Brussel
